# Aldo-keto reductases are biomarkers of NRF2 activity and are co-ordinately overexpressed in non-small cell lung cancer

**DOI:** 10.1038/bjc.2016.363

**Published:** 2016-11-08

**Authors:** A Kenneth MacLeod, Lourdes Acosta-Jimenez, Philip J Coates, Michael McMahon, Frank A Carey, Tadashi Honda, Colin J Henderson, C Roland Wolf

**Affiliations:** 1 Division of Cancer Research, School of Medicine, University of Dundee, Ninewells Hospital, Dundee DD1 9SY, UK; 2 Department of Pathology and Neuroscience, Ninewells Hospital and Medical School, Dundee DD1 9SY, UK; 3 Department of Chemistry and Institute of Chemical Biology and Drug Discovery, Stony Brook University, Stony Brook, NY 11794-3400, USA

**Keywords:** lung cancer, biomarkers, stress response, NRF2, aldo-keto reductase

## Abstract

**Background:**

Although the nuclear factor-erythroid 2-related factor 2 (NRF2) pathway is one of the most frequently dysregulated in cancer, it is not clear whether mutational status is a good predictor of NRF2 activity. Here we utilise four members of the aldo-keto reductase (AKR) superfamily as biomarkers to address this question.

**Methods:**

Twenty-three cell lines of diverse origin and NRF2-pathway mutational status were used to determine the relationship between AKR expression and NRF2 activity. AKR expression was evaluated in lung cancer biopsies and Cancer Genome Atlas (TCGA) and Oncomine data sets.

**Results:**

AKRs were expressed at a high basal level in cell lines carrying mutations in the *NRF2* pathway. In non-mutant cell lines, co-ordinate induction of AKRs was consistently observed following activation of NRF2. Immunohistochemical analysis of lung tumour biopsies and interrogation of TCGA data revealed that AKRs are enriched in both squamous cell carcinomas (SCCs) and adenocarcinomas that contain somatic alterations in the NRF2 pathway but, in the case of SCC, AKRs were also enriched in most other tumours.

**Conclusions:**

An AKR biomarker panel can be used to determine NRF2 status in tumours. Hyperactivation of the NRF2 pathway is far more prevalent in lung SCC than previously predicted by genomic analyses.

NRF2 is a cap‘n'collar (CNC) basic-region leucine zipper (bZIP) transcription factor that regulates a diverse battery of cytoprotective genes that collectively allow cells to survive transient periods of exposure to electrophilic, oxidative and inflammatory stress (Itoh *et al*, 1997; Hayes *et al*, 2010). The stability, abundance and activity of NRF2 is primarily governed by kelch-like ECH-associated protein 1 (KEAP1), a homodimeric substrate adaptor that recruits the factor to the Cullin 3/Ring-box 1 (CUL3/RBX1) E3 ubiquitin ligase holoenzyme, targeting it for ubiquitylation and subsequent proteasomal degradation (Itoh *et al*, 1999; McMahon *et al*, 2003). This targeting is inhibited by toxic electrophiles, such as lipid peroxidation products or the reactive metabolites of chemicals or therapeutic drugs, which directly modify reactive cysteine residues on KEAP1, leading to nuclear accumulation of NRF2 and increased transcription of cytoprotective genes (Dinkova-Kostova *et al*, 2002).

The increased capacity to inactivate genotoxic electrophiles conferred by activation of NRF2 can reduce the rate at which normal cells accumulate mutations, and can thereby inhibit chemically mediated carcinogenesis in animal models. Accordingly, Nrf2-null mice are more susceptible to this process (Ramos-Gomez *et al*, 2001). However, following tumorigenesis, NRF2 may have a role in the malignant progression of lung adenoma to adenocarcinoma as the incidence and malignant characteristics of tumours in wild-type animals are greater than those in Nrf2-knockout animals (Satoh *et al*, 2013). Generally, once a cancer has emerged, increased NRF2 activity is thought to promote cell survival and proliferation under conditions of environmental stress or in the face of chemotherapy (Hayes and McMahon, 2009). Consistent with this, genetic, epigenetic and signalling changes in tumours that exacerbate NRF2 activity have been associated with poor patient outcome (Shibata *et al*, 2008b). For this reason, NRF2 inhibition has emerged as a potential chemotherapeutic strategy.

Research into NRF2 has been impeded by the lack of sufficiently specific and sensitive antibodies. Its abundance is often, therefore, inferred from target gene/protein expression levels. This target gene battery is well defined in mouse, but is not so thoroughly characterised in man. Microarray and ChIP-Seq analyses of mouse cells have established that the CNC/bZIP factor is involved in the transcriptional regulation of several hundred genes (Thimmulappa *et al*, 2002; Malhotra *et al*, 2010). In human cell lines, a number of studies have identified the transcriptomic and, in some cases, proteomic changes that arise upon chemical or genetic perturbation of NRF2 signalling (MacLeod *et al*, 2009; Agyeman *et al*, 2012). Much of the NRF2 target gene battery is conserved between mouse, rat and man. Common targets include genes involved in antioxidant processes, NADPH generation, metal binding and the stress response (Hayes *et al*, 2010). There is divergence, however, in the drug metabolism enzyme targets of NRF2. In mouse, glutathione *S*-transferases (GSTs) predominate, while the aldo-keto reductases (AKRs) are more conspicuous when human cells are examined. AKR1B10, AKR1C1, AKR1C2 and AKR1C3 constitute some of the most inducible targets of NRF2 in human systems, both normal and tumour-derived (Lou *et al*, 2006; MacLeod *et al*, 2009; Agyeman *et al*, 2012; Jung *et al*, 2013). The DNA motif bound by NRF2, the antioxidant response element (ARE), has been identified in the promoters of *AKR1B10*, *AKR1C1* and *AKR1C2* (Lou *et al*, 2006; Nishinaka *et al*, 2011).

In order to examine whether the ability of NRF2 to drive *AKR* gene transcription is ubiquitous in human cell types of different origin, we have measured the expression of mRNA and protein for these targets in a panel of cultured cell lines (mostly tumour-derived) in both basal and induced states. We show that AKR expression is related to the status of the NRF2/KEAP1 pathway and can be used as a readout for the activation or inhibition of NRF2. Through immunohistochemical analysis of lung tumour biopsies and interrogation of data from The Cancer Genome Atlas (TCGA), we have found that AKRs are enriched in both SCC and AC that contain somatic alterations in the NRF2 pathway but, in the case of SCC, AKR enrichment also occurs in cells not carrying *NRF2* or *KEAP1* mutation. These data indicate that NRF2 is frequently constitutively activated by alternative mechanisms in this cancer type and, therefore, that genetic analyses alone cannot determine the contribution of NRF2 to the tumour phenotype.

## Materials and methods

### Cell lines

A panel of cell lines containing either wild type or mutant forms of *NRF2* or *KEAP1*, as confirmed by the Wellcome Trust Sanger Institute COSMIC database, was assembled. The origin, authentication and culture conditions of these cell lines are described in detail in Supplementary Materials and Methods. Characteristics of the mutant cell lines are detailed in Supplementary Table 1. All lines were free of mycoplasma contamination, as verified using the MycoAlert Mycoplasma detection kit (Lonza, Basel, Switzerland). In contrast to previous reports (Singh *et al*, 2006), the Wellcome Trust Sanger Institute COSMIC database states that the H23 cell line contains a homozygous mutation in *KEAP1* (579G>C, Q193H), while neither H1395 nor H1993 contain the reported heterozygous mutation (both 1048G>A, G350S). Our sequencing of these cell lines agreed with the Sanger database entries in each case (Supplementary Figure 1).

### Cell treatments, sample preparation and ELISA

Sulforaphane (SFN) was purchased from LKT laboratories (St Paul, MN, USA). (±)-(4a*α*, 8a*α*, 10a*β*)-1, 2, 4a, 6, 8a, 9, 10, 10a-octahydro-8a-ethynyl-1,1,4a-trimethyl-2,6-dioxophenanthrene-3,7-dicarbonitrile (TBE-31) was synthesised as described previously (Honda *et al*, 2007; Saito *et al*, 2013). Cells were treated with 5 *μ*mol l^−1^ SFN or 0.2 *μ*mol l^−1^ TBE-31 in 0.1% acetonitrile vehicle on reaching 50–70% confluency, and protein or cDNA samples prepared 24 h later. For knockdown experiments, A549 and H838 cells were reverse-transfected with ON-TARGET*plus* NRF2 (L-003755-00) and non-targeting (D-001810-10-05) siRNA SMARTpools, each containing four siRNAs (Dharmacon, Thermo Fisher Scientific, Waltham, MA, USA), at a final concentration of 10 nmol l^−1^, in complex with Lipofectamine RNAiMAX (Life Technologies, Carlsbad, CA, USA). Cells were lysed for analysis at the timepoints indicated. For fractionation of nuclear and cytoplasmic compartments, cells were processed using the NE-PER Nuclear and Cytoplasmic Extraction kit (Thermo Fisher Scientific). ELISAs were carried out by the In-Cell colorimetric method (Thermo Fisher Scientific) and statistical significance evaluated by unpaired *t*-test: *P⩽0.05, **P⩽0.01, ***P⩽0.001.

### Antibodies and western blotting

Rabbit polyclonal anti-AKR1B10, anti-AKR1C1, anti-NQO1, anti-KEAP1 and anti-NRF2 were kindly provided by Professor John Hayes, University of Dundee. Mouse polyclonal anti-AKR1C1 (ab72576), mouse monoclonal anti-GAPDH (ab8245), mouse monoclonal anti-TBP (ab51841) and rabbit monoclonal anti-LDH (ab52488) were purchased from Abcam (Cambridge, UK). Mouse monoclonal anti-AKR1C3 was a kind gift from Professor Trevor Penning, University of Pennsylvania, PA, USA. SDS–PAGE and western blotting were carried out as described previously (MacLeod *et al*, 2009).

### Measurement of mRNA levels

mRNA analysis was carried out in 96-well plate format using Applied Biosystems' TaqMan Gene Expression Cells-to-CT kit (Life Technologies) according to the manufacturer's instructions. All real-time PCR primer and probe sets were purchased from Applied Biosystems (Foster City, CA, USA); AKR1B10 (Hs00252524_m1), AKR1C1/2 (Hs00413886_m1), AKR1C3 (Hs00366267_m1), NRF2 (Hs00975960_m1), Actin B internal standard (4352935E). Assays were performed in triplicate and fold changes calculated using the 2(−δδ C(T)) method. Fold changes for inducer-treated cells were calculated relative to vehicle-treated cells and the heatmap generated using ggplot2 in R. Statistical significance was evaluated using an unpaired *t*-test. For Figures 3B and C: *P⩽0.05, **P⩽0.01, ***P⩽0.001. For Supplementary Table 2, FDR (Q) was set to 1% and significant differences are denoted ‘*'.

### TCGA data retrieval and analysis

All gene expression and mutation data for AC and SCC were derived from publicly available TCGA datasets (15 July 2015, https://tcga-data.nci.nih.gov/tcga/). For AC, analyses were performed on the ‘TCGA, Nature 2014' data set. For SCC, analyses were performed on the ‘TCGA Provisional' data set (mutation information from samples shared with the ‘TCGA, Nature 2012' data set). Normalised level 3 RNA-seq gene expression values, as determined using the RNA-Seq by Expectation Maximisation (RSEM) algorithm, were retrieved using the TCGA-Assembler package in R and RStudio (Zhu *et al*, 2014). Mutation data for *NRF2* and *KEAP1* were retrieved using the cBioPortal web application programming interface (www.cbioportal.org). For the comparison of wild type with mutant non-small cell lung cancer (NSCLC), we analysed 230 cases of AC (179 wild type, 51 mutant) and 178 cases of SCC (120 wild type, 58 mutant). For the comparison of tumour to matched normal tissue we analysed data for 45 tumour/normal (35 wild type, 10 mutant) paired AC and 16 tumour/normal (11 wild type, 5 mutant) paired SCC samples. All data processing and statistical analyses were carried out in R using packages cgdsr, plyr and ggplot2. Association was evaluated by unpaired and paired Wilcoxon ranked test for wild-type *vs* mutant values (Figure 4) and matched normal *vs* tumour values (Figure 5), respectively.

## Results

### NRF2 or KEAP1 mutation leads to a high basal level of AKR1B, AKR1C1/2 and AKR1C3 expression

**Figure 1:**
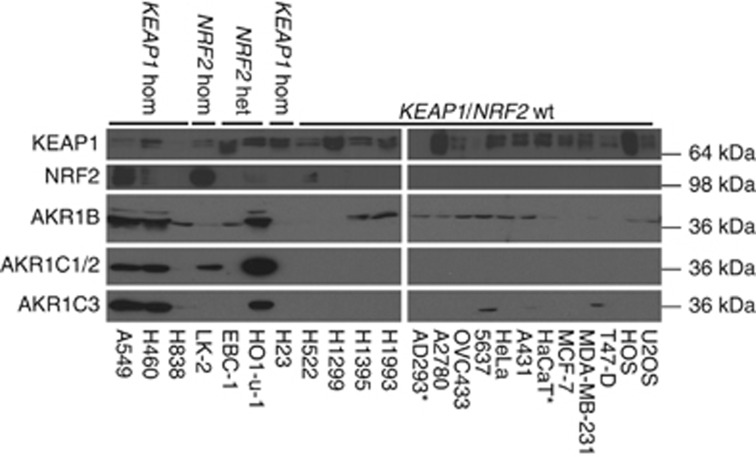
**AKR1B, AKR1C1/2 and AKR1C3 are expressed at high levels in cell lines carrying mutant *KEAP1* or *NRF2*.** Western blotting of whole-cell lysates for AKR1B, AKR1C1/2 and AKR1C3 was carried out as described in Materials and Methods. Samples were prepared and analysed in parallel and uniformity of sample loading on the gels was verified by Coomassie blue staining (data not shown). *NRF2 and *KEAP1* mutational status in AD293 and HaCaT cell lines unknown.

Whole-cell lysates were prepared from a panel of cell lines, some of which carry mutations in *NRF2* or *KEAP1*, and AKR protein expression measured by western blotting (Figure 1). The rabbit antibody raised against AKR1B10 (36.02 kDa) also reacts with AKR1B1 (35.85 kDa). Similarly, as AKR1C1 (36.79 kDa) and AKR1C2 (36.74 kDa) proteins are 98% identical, it is likely that the mouse polyclonal antibody used to detect AKR1C1 cross-reacts with AKR1C2. The mouse monoclonal antibody used to detect AKR1C3 is highly specific; cross-reactivity with recombinant forms of human AKR1A, AKR1B, AKR1C and AKR1D superfamily members was not observed (data not shown).

AKR1B, AKR1C1/2 and AKR1C3 were all expressed constitutively at the highest levels in A549, H460 (both of which are homozygous for mutations in *KEAP1*) and HO1-u-1 (heterozygous for *NRF2* mutation, Figure 1). In addition, AKR1C1/2 was also highly expressed in LK-2 cells (homozygous for *NRF2* mutation), while AKR1C3 was expressed at low, but detectable, levels in H838 (homozygous for *KEAP1* mutation), 5637 and MDA-MB-231 (*KEAP1* and *NRF2* wild type) cells (Figure 1). It should be noted that none of the cell lines possess mutant CUL3, with the exception of H460 (1299C>T, T410I), which also carries mutant KEAP1 (Supplementary Table 1).

### AKR1B, AKR1C1/2 and AKR1C3 are inducible in cell lines with functional, but not mutated, KEAP1/NRF2

**Figure 2:**
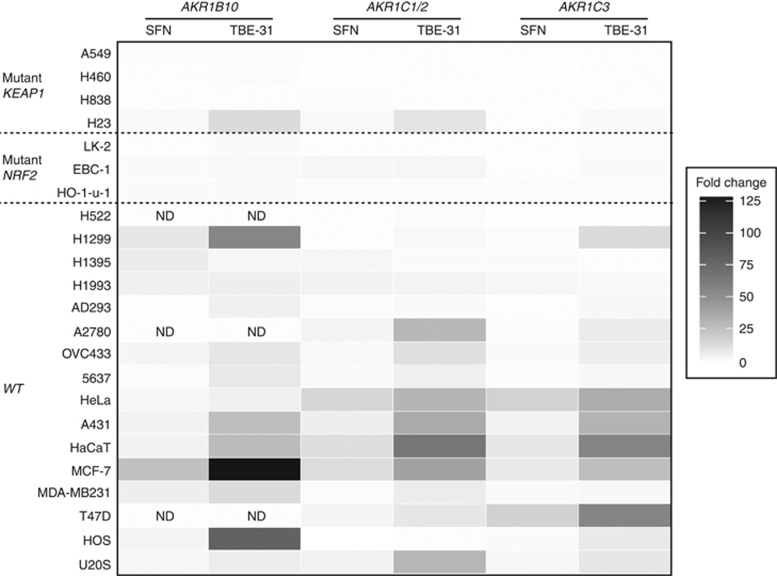
**Chemical activators of NRF2 induce AKR mRNA in human cell lines with wild-type *KEAP1* and *NRF2*.** Cells were seeded, incubated under standard conditions for 24 h, then treated with 5 *μ*mol l^−1^ SFN, 0.2 *μ*mol l^−1^ TBE-31 or vehicle control. After a further 24 h, cells were lysed, cDNA synthesised, and RT–PCR carried out for AKR1B10, AKR1C1/2 and AKR1C3. Fold change values for SFN and TBE-31-treated cells were calculated relative to vehicle control cells. ND: mRNA for *AKR1B10* was not detected in H522, A2780 and T47D cells treated with vehicle, preventing accurate quantification of fold change.

Cell lines were treated for 24 h with two NRF2 inducers; SFN, an isothiocyanate and a widely used activator of NRF2, and TBE-31, a highly potent tricyclic-*bis*-enone inducer (Honda *et al*, 2007; Liby *et al*, 2008). Remarkably, Taqman analysis of RNA indicated that SFN and TBE-31 induced *AKR1B10*, *AKR1C1/2* and *AKR1C3* mRNA in almost all tumour cell lines with wild-type *KEAP1* and *NRF2*, independent of the tissue or origin (Figure 2, fold change values are provided in Supplementary Table 2). At the concentrations tested, fold induction in response to TBE-31 was generally much greater than in response to SFN. The presence of two cyano enone moieties within TBE-31 have previously been shown to underpin this high relative potency. With the exception of H23, AKRs were not inducible in cell lines with biallelic mutations in *KEAP1* or *NRF2*. In one cell line carrying heterozygous *NRF2* mutation, EBC-1, *AKR* mRNAs could still be modestly upregulated in response to inducer treatment.

**Figure 3:**
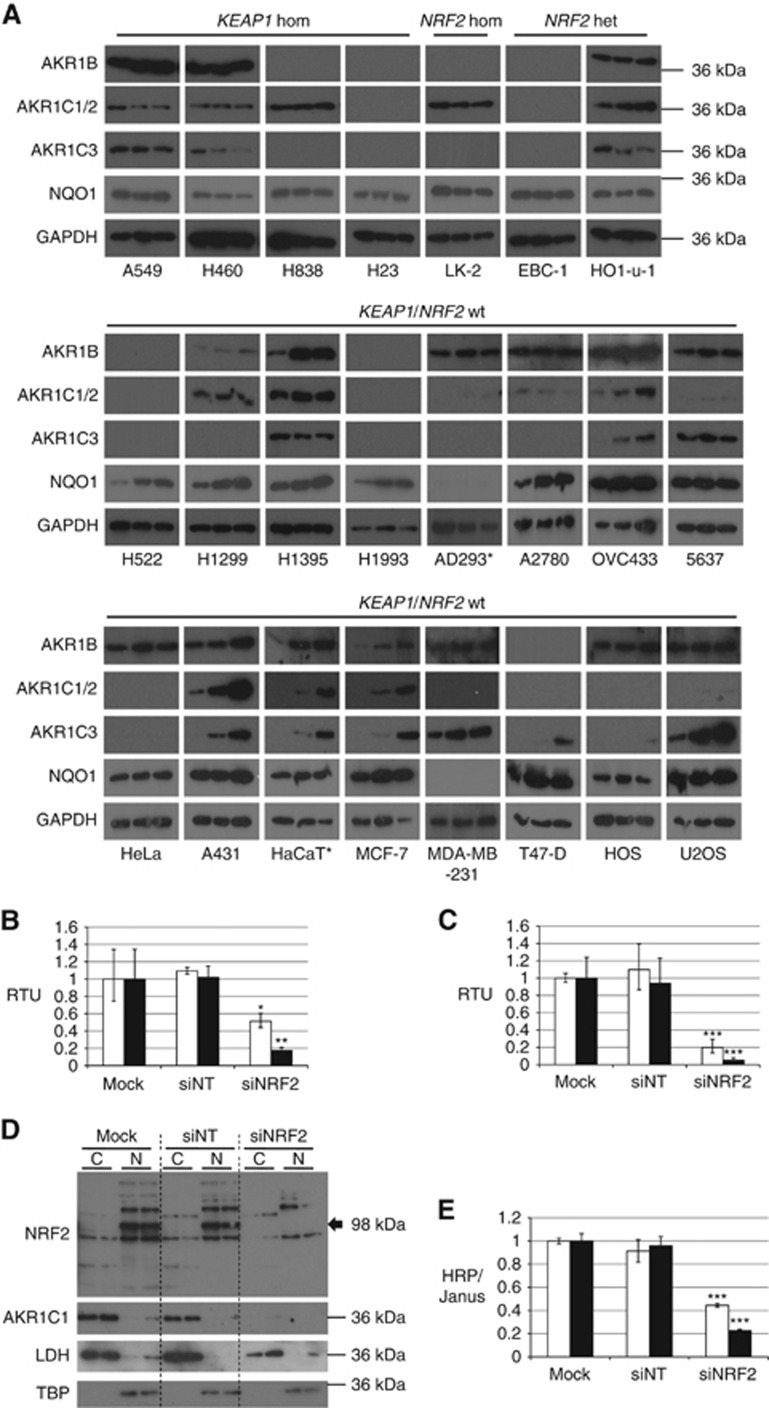
**Constitutive and inducible expression of AKR proteins in human cell lines.** (**A**) Chemical activators of NRF2 induce AKR protein in human cells possessing wild-type *KEAP1*/*NRF2*. Cells were seeded and incubated under standard conditions for 24 h. Medium was replaced with fresh complete medium containing inducer compounds in acetonitrile (final concentration of acetonitrile, 0.1% (v/v)), then incubated for a further 24 h. Protein samples were prepared from whole-cell lysates and immunoblotted for AKR1B, AKR1C1/2, AKR1C3 and NQO1. In each blot, lane 1 contains acetonitrile vehicle control, lane 2 contains 5 *μ*mol l^−1^ SFN and lane 3 contains 0.2 *μ*mol l^−1^ TBE-31. Data are representative of two separate experiments. *NRF2 and *KEAP1* mutational status in AD293 and HaCaT cell lines unknown. (**B**–**E**) Knockdown of NRF2 leads to decreased AKR1C1/2 expression in A549 and H838 cells. TaqMan RT–PCR was carried out for NRF2 (white bars) and AKR1C1 (black bars) in A549 cells (**B**) and H838 cells (**C**) 48 h after mock reverse transfection (transfection reagent alone), or reverse transfection with siRNA. RTU, relative transcription units. (**D**) Western blots were carried out for NRF2 (black arrow) and AKR1C1 in A549 cells 120 h after reverse transfection. Lactate dehydrogenase (LDH) and TATA binding protein (TBP) blots are shown to demonstrate effective separation of the cytoplasmic and nuclear fractions, respectively. (**E**) In-Cell ELISA of AKR1C1 was carried out 120 h after reverse transfection in A549 (white bars) and H838 (black bars) cells. HRP (horseradish peroxidase) signal was adjusted for cell density using Janus Green cell stain. For **B**, **C** and **E**, statistical significance was calculated relative to mock-transfected control cells. *P⩽0.05, **P⩽0.01, ***P⩽0.001.

In many of the cell lines homozygous for wild-type *KEAP1* and *NRF2*, the induction of *AKR* mRNA was paralleled by an increase in protein (Figure 3A, summarised in Supplementary Table 3). In some cell lines, all targets were induced (for example, OVC433, A431, HaCaT and MCF-7 cells) while, in others the induction was much less marked. The western blots shown in Figure 3A were exposed for varying lengths of time in order to optimise chemiluminescent signal intensity to determine AKR induction on a cell line basis. The data should therefore not be compared directly with Figure 1. In many cases, AKR expression was close to or below the limit of detection with the blotting protocol applied, preventing a full comparison with the mRNA data. No induction of AKR proteins was detected in any of the *KEAP1*/*NRF2* mutant cell lines, with the exception of AKR1C1/2 in HO1-u-1 cells. Western blotting for NAD(P)H dehydrogenase, quinone 1 (NQO1), the protein most commonly used as a proxy measure of NRF2 activity, showed that it was constitutively expressed in almost all of the cell lines, being undetectable in only AD293 and MDA-MB-231. NQO1 was inducible in many of the lines homozygous for wild-type *KEAP1* and *NRF2*, but the fold changes were less than those of the AKRs. NQO1 was not inducible in the mutant cell lines.

### Knockdown of NRF2 in KEAP1 homozygous mutant cells leads to decreases in AKR1C1 expression

The A549 and H838 cell lines, which lack functional KEAP1 and hence exhibit elevated NRF2 activity (Singh *et al*, 2006), were transfected with either non-targeting siRNA (siNT), or siRNA against NRF2 (siNRF2). In both cell lines, NRF2 knockdown reduced the mRNA level to between 20 and 50% of control level (Figure 3B), with a concomitant decrease in the level of AKR1C1/2 mRNA to ∼5–20% of control, after 48 h (Figure 3C). Protein levels for both NRF2 and AKR1C1/2 were decreased in A549 cells, and this effect was maintained up to 120 h (Figure 3D). AKR1C1/2 depletion after 120 h was also observed by the In-Cell ELISA method, using mouse polyclonal anti-AKR1C1, in both A559 and H838 cells (Figure 3E). The proportion of decrease in AKR1C1/2 expression is similar to that of other NRF2 targets, as described in previous studies (Singh *et al*, 2006, 2008).

### AKR1B, AKR1C1/2 and AKR1C3 are co-ordinately upregulated in the majority of lung SCC and in the minority of lung AC

Rabbit polyclonal anti-AKR1B10 and anti-AKR1C1 and mouse monoclonal anti-AKR1C3 were used for immunohistochemistry. All three of these primary antibody preparations give single bands on western blot of whole-cell lysate protein samples from A549 cells, indicating their specificity (Supplementary Figure 2). We initially determined whether the antibodies were suitable for immunohistochemistry by staining FFPE preparations of cultured cell lines; the MCF-7-derived AREc32 cell line, in which NRF2 activity is inducible, and the A549 cell line, in which NRF2 activity is high due to a homozygous inactivating mutation in *KEAP1* (Singh *et al*, 2006). The anticipated pattern of expression was observed, wherein AREc32 cells show minimal staining that is increased following treatment with 5 *μ*M SFN for 24 h, and A549 cells show the strongest staining, reflecting their *KEAP1* mutant status (Supplementary Figure 3).

Fifteen FFPE samples of NSCLC were classified by conventional histological criteria as either SCC (*n*=8) or AC (*n*=7). Positive immunohistochemical staining was detected for at least one AKR in all SCC samples and for two or three AKRs in seven of eight samples (scoring provided in Table 1, representative images shown in Supplementary Figure 4). Staining was observed for all three AKRs in two of seven AC, while no staining was detected in the remaining five. The difference between SCC and AC was found to be statistically significant (*P*=0.0002) by two-tailed Fisher's exact test.

### AKR mRNA is highly enriched in KEAP1/NRF2 mutant AC and SCC, but also in KEAP1/NRF2 wild-type SCC

**Figure 4:**
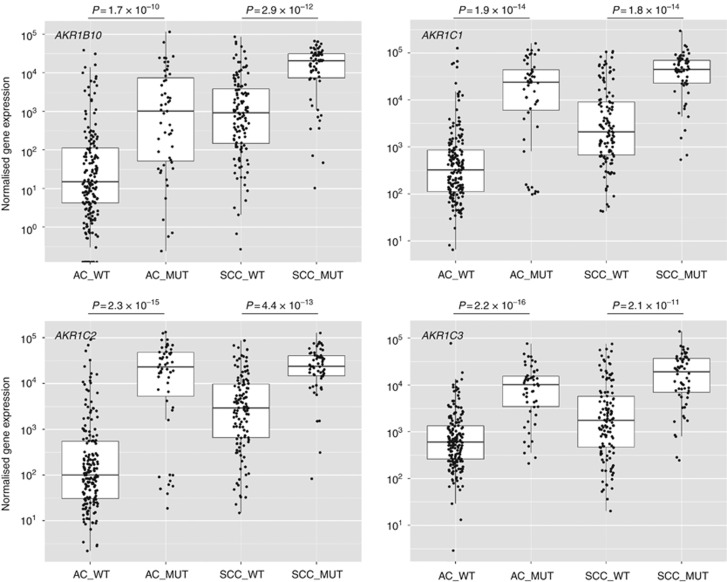
***AKR* mRNA is upregulated in both *KEAP1*/*NRF2* mutant AC and SCC.**TCGA data for NSCLC were processed and analysed as described in Materials and Methods. Both AC and SCC cases were defined as mutant (MUT) if they possessed one or more of the following: somatic mutation of *KEAP1*, loss of heterozygosity of *KEAP1*, somatic mutation of *NRF2*, gene amplification of *NRF2*.

Data were retrieved from TCGA for NSCLC as described in Materials and Methods. A comparison of samples wild type or mutant for the NRF2 pathway demonstrated a highly significant enrichment of mRNA for all four *AKR* genes in mutant cases, in both AC and SCC (Figure 4). In each of the *AKR1C1* and *AKR1C2* plots, we noted what appeared to be a distinct cluster of mutant AC (16% and 18% of the total mutant population, respectively) that had low expression of the transcript, approximately equal to the wild-type median level. Case IDs for the 10 samples with the lowest level of expression of each of the four genes under study were compared (Supplementary Table 4). There was a high degree of similarity between the lists indicating that if one of the transcripts was expressed at a low level it was likely that the other three were also expressed at a low level. These cases contained a variety of the specified mutational types: segregation to the low-expression cluster was not dependent on only one type of pathway alteration. Moreover, when *AKR* data from SCC were assessed for co-expressed genes in cBioPortal (www.cbioportal.org), strong correlations were observed between *AKR*s, and of *AKR*s with other NRF2-regulated genes, such as *GPX2*, *GSR*, *PGD*, *G6PD*, *GCLM*, *GCLC*, *ME1*, *SRXN1* and *NQO1* (Supplementary Table 5). There was no association of *CUL3* mutation status with *AKR* mRNA level in either AC or SCC when all samples were considered (Supplementary Figure 5), although there was a statistically significant increase in *AKR1B10*, *AKR1C1* and *AKR1C2* mRNA in SCC when cases which were mutant for *KEAP1* and/or *NRF2* were removed from the wild-type population (Supplementary Figure 6).

**Figure 5:**
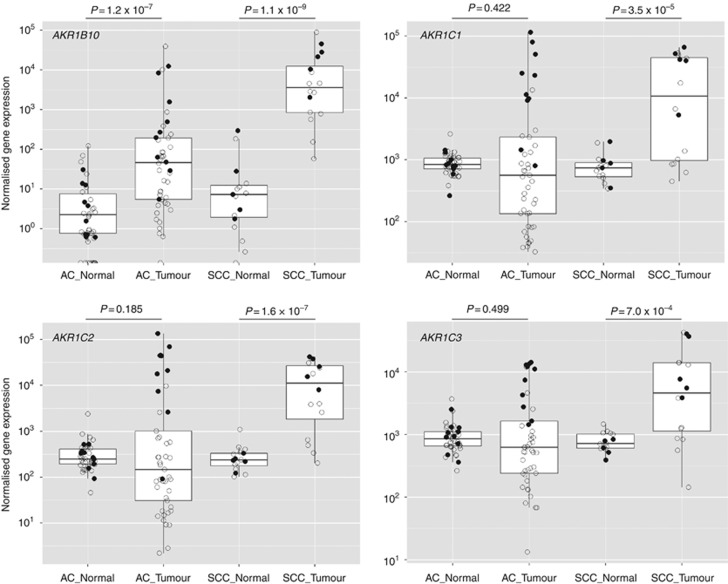
**Induction of AKR mRNA is more widespread in SCC than in AC, irrespective of *KEAP1*/*NRF2* mutation status.** Paired normal/tumour sample data from TCGA were processed and analysed as described in Materials and Methods. Cases in which the tumour was either wild type (open circles) or mutant (closed circles) in respect to *KEAP1*/*NRF2* mutation status are shown. Statistical significance of *AKR* enrichment relative to normal tissue is calculated as a combined score for both wild-type and mutant cases.

We compared matched tumour/normal samples from 51 cases of AC and 53 cases of SCC. For AC, all four *AKR*s showed a wider range of expression in tumour tissue than in normal tissue (Figure 5). This spread of values was largely associated with higher *AKR* levels in *NRF2*/*KEAP1* mutant cases. Overall, the median level of *AKR1C1*, *AKR1C2* and *AKR1C3* was decreased in tumour, relative to normal tissue but when only mutant cases were considered, all AKRs were significantly increased. Paired Wilcoxon ranked test determined *P*-values of tumour *vs* normal gene expression for matched mutant AC samples (*n*=10) as follows; *AKR1B10*: 0.002, *AKR1C1*: 0.002, *AKR1C2*: 0.004, *AKR1C3*: 0.002. In SCC, all four *AKR*s were highly significantly enriched in tumour tissue, compared with normal, irrespective of mutational status, although mutant cases generally possessed a higher relative level of expression. Median fold enrichments in SCC were as follows: *AKR1B10*: 529-fold, *AKR1C1*: 16-fold, *AKR1C2*: 50-fold, *AKR1C3*: 7-fold. This pattern of enrichment in AC and SCC was also seen with the other NRF2 target genes, *GCLC*, *GCLM* and *NQO1*, although *HMOX1* was downregulated in both tumour types (Supplementary Figure 7).

The association of *AKR1B10* mRNA expression with tumour type was investigated in separate microarray data sets using the Oncomine platform (www.oncomine.org). We found a highly significant association of *AKR1B10* overexpression with SCC of the lung in multiple analyses; examples from Lee *et al* (2008) and Zhu *et al* (2010) are presented in Supplementary Figure 8. As with TCGA data, expression of *AKR1B10* correlated with that of *AKR1C1*, *AKR1C2*, *AKR1C3* and other targets of NRF2, such as *GPX2*, *GSR*, *PGD* and *G6PD*, with widespread co-induction in SCC (examples from Lee *et al* (2008) and Zhu *et al* (2010) are shown in Supplementary Figure 9).

## Discussion

The NRF2 pathway may become activated in tumours through a large number of mechanisms. Core components of the pathway itself are some of the most frequently mutated genes in cancer, with mutations in *NRF2* and *KEAP1* occurring in diverse tumour types (Singh *et al*, 2006; Shibata *et al*, 2008a, 2008b; Kim *et al*, 2010; Zhang *et al*, 2010; Yoo *et al*, 2012). *NRF2* may be transcriptionally upregulated by oncogenic forms of K-Ras, B-Raf and Myc (Denicola *et al*, 2011), although it should be noted here that we did not find a correlation between mutation in these genes and enrichment for AKR transcripts (cBioPortal, data not shown). Alternatively, NRF2 activity may increase in response to stimuli of exogenous origin, such as chemotherapeutic drugs (McMahon *et al*, 2014) or dietary compounds. KEAP1 levels may be decreased through promoter methylation (Martinez *et al*, 2013) or by microRNA-mediated interference (van Jaarsveld *et al*, 2013), or the protein itself may be chemically inactivated (Ooi *et al*, 2011). Mutation or deletion of CUL3, or methylation of its promoter, have been reported to occur at high frequencies in certain cancers (CGARN, 2012; Martinez *et al*, 2013). While CUL3 is undoubtedly a key regulator of NRF2 status, its interaction with a large number of substrate adaptor proteins facilitates the ubiquitination of many targets. Indeed, mutation of *CUL3* did not correlate with increased expression of NRF2 target genes in the TCGA data sets, and actually tended towards co-occurrence with mutations in *KEAP1* and *NRF2* in SCC (cBioPortal, data not shown). We therefore disregarded this factor in our analyses to maximise specificity for the NRF2 pathway.

The implication of these (and other) observations is that constitutive NRF2 activity is far more common in tumours than has been predicted by the frequency of genomic or epigenomic alteration of the pathway, and that these analyses cannot therefore definitively predict its phenotypic status. In order to effectively interrogate CUL3/KEAP1/NRF2 pathway status, a robust downstream signature of activity is required. We had previously identified AKRs 1B10, 1C1, 1C2 and 1C3 as some of the most inducible targets of the human NRF2 pathway in the spontaneously immortalised keratinocyte cell line, HaCaT (MacLeod *et al*, 2009), a finding since replicated in non-tumorigenic MCF10A and MCF12A breast epithelial cells, virally-immortalised HK-2 kidney cells and the U937 lymphoma cell line (Agyeman *et al*, 2012; Jung *et al*, 2013). Our aim in the present study was to determine the extent of this relationship across a large panel of cell lines and tumour samples.

We found that, generally, cultured cell lines are either responsive to inducers of NRF2, or have mutations in the pathway. In 23 cell lines of diverse origin we observed high constitutive levels of expression of AKR1B, AKR1C1/2 and AKR1C3 in three (A549, H460, HO1-u-1) of seven lines carrying mutant *KEAP1* or *NRF2*. Further, two (H838, LK-2) of the remaining mutant lines exhibited high levels of one these enzymes individually. High levels of AKR coincided with high levels of NRF2. With very few exceptions (AKR1B in H1395 and H1993, and AKR1C3 in 5637 and MBA231), AKRs were expressed at relatively low constitutive levels in cell lines with non-mutated *KEAP1*/*NRF2*. Importantly, the true (relative) level of NRF2 activity in each of these cell lines is unknown. It has been shown, however, that certain *KEAP1* mutations have little or no influence on the capacity of KEAP1 to repress NRF2 (Hast *et al*, 2014), which may explain why the H838 and H23 cell lines can carry mutant *KEAP1* but express relatively low (compared with A549, H460 and HO-1-u-1) basal levels of AKR. In the case of H838, it has previously been shown that these cells exhibit lower levels of enzymatic activity of NQO1, the most commonly used proxy for NRF2, than do A549 and H460 (Singh *et al*, 2006). In the case of H23, these cells apparently retain the capacity for NRF2-mediated induction of AKRs, implying that the KEAP1/NRF2 axis is functional. Regarding the *NRF2*-mutant cell lines, and in agreement with our data, Shibata *et al* (2008b) reported that NRF2-driven luciferase activity in EBC1 cells was more responsive than that in LK2 cells to ectopic expression of KEAP1, suggesting that, as with KEAP1, mutations in NRF2 are not functionally equivalent. In our analysis of TCGA data, we observed a strong association of NRF2 pathway mutational status with *AKR* mRNA levels, in agreement with the observations of Cescon *et al* (2015). Interestingly, we observed a subgroup of *KEAP1*/*NRF2* mutant AC that did not show enrichment for *AKR* mRNA. Conversely, we found that many cases of SCC in which *KEAP1* and *NRF2* were wild type exhibited higher levels of AKR expression than their matched normal controls. Importantly, these observations suggest that mutation in the *KEAP1*/*NRF*2 pathway and high levels of target gene expression are not interchangeable descriptors, and that a biomarker signature, such as that described here, provides a more definitive measure of NRF2 activation relative to a proxy measure such as genotype/epigenotype. Furthermore, the assessment of AKR levels potentially constitutes a means to discriminate between the relative functionality of *KEAP1* and *NRF2* mutations and, ultimately, whether they are likely to influence tumour phenotype.

As Phase I drug metabolism enzymes, AKRs have been implicated in the bioactivation and detoxification of chemotherapeutic drugs (Jin and Penning, 2007). Upregulation of AKR expression by NRF2 could therefore alter chemoresponse, with a potentially deleterious change in efficacy and/or toxicity. Overexpression of AKR1Cs has been identified as a mechanism of resistance to several commonly used agents, for example platinum compounds (Deng *et al*, 2004). AKRs have also been implicated in the genesis of cancer. Two distinct pro-proliferative functions have been identified for AKR1B10. First, this enzyme converts retinal to retinol, thereby preventing the generation of retinoic acid and consequently alleviating the anti-proliferative effect of this metabolite (Tang and Gudas, 2011). Second, AKR1B10 reduces farnesal and geranylgeranyl (Endo *et al*, 2009), a key step in the process of protein prenylation which is required by KRAS (and other oncoproteins) for their function. Consistent with this latter role, chemical inhibition of AKR1B10 was shown to inhibit carcinogenesis in a Kras^G12D^/Trp53^R172H^ mouse model of pancreatic cancer, with an accompanying decrease in Ras signalling (Li *et al*, 2013). Moreover, this function may also, at least in part, explain why knockout of Nrf2 inhibits the genesis and proliferation of pancreatic and lung Kras^G12D^ tumours (Denicola *et al*, 2011). Of particular relevance to smokers, AKR enzymes metabolically activate polycyclic aromatic hydrocarbons, converting them to electrophilic and redox-active o-quinones (Palackal *et al*, 2002). These same enzymes are upregulated in response to cigarette smoke, indicating a further potential mechanistic contribution of NRF2 dysregulation to tumour genesis and promotion in this demographic (Zhang *et al*, 2008). Finally, and possibly relating to the hormonal metabolism functions of enzymes of the AKR1C subfamily, the enzymatic activity of ectopically expressed AKR1C1 and AKR1C2 has been shown to enhance tumourigenesis of NIH3T3 xenografts in mice (Chien *et al*, 2009). Collectively, these findings implicate AKRs in the genesis of cancer, extending their involvement beyond pathways of xenobiotic metabolism. This potential role is further evidenced by our demonstration of the ubiquitous nature of the NRF2/AKR relationship.

Upon challenge with chemical inducers of NRF2, we observed an increase in mRNA for *AKR1B10*, *AKR1C1*/*2* and *AKR1C3* in almost all cell lines containing non-mutated *KEAP1* and *NRF2*, whereas cell lines carrying heterozygous mutations in these genes were less responsive to these compounds and, with the exception of H23, homozygous mutant cell lines were completely non-responsive. These findings strongly support the contention that AKRs are co-regulated as part of the NRF2 gene battery and, moreover, indicate their potential utility for inferring NRF2 hyperactivation in tumour tissue, irrespective of its mechanistic origin. Interestingly, AKRs were more markedly induced than NQO1 following chemical activation of NRF2 in wild-type cells, which is significant because NQO1 is currently the most widely used biomarker of NRF2 activation. This contrasts somewhat with the TCGA analysis, in which *NQO1* performed similarly to *AKR*s. (Notably, *HMOX1*, another commonly used proxy for NRF2 levels, did not show any indication of differential expression in tumours, mutant or otherwise.) As all NRF2 target genes will be subject to multifactorial regulation, we suggest that the expression of AKRs should be considered a signature of NRF2 activity. This signature, while generally segregating with *NRF2*/*KEAP1* genotype in cultured cells (i.e., higher expression in mutant cells), did not appear to do so in lung SCC, as high levels of AKR were detected in tumours without these mutations. This finding suggests that additional mechanisms of pathway regulation, potentially those with roots in the environmental or stromal context of the tumour, may often be key determinants of its status *in vivo*. In addition, our data support the contention that, while the transient activation of this pathway may be chemopreventive in normal cells, its chronic activation in cancer cells confers a selective advantage through enhanced proliferation and chemoresistance.

Non-small cell lung cancer is the tumour type in which KEAP1/NRF2 dysfunction has most frequently been reported. Within NSCLC, mutations in *KEAP1* occur primarily in AC and mutations in *NRF2* occur primarily in SCC (Singh *et al*, 2006; Shibata *et al*, 2008b; Hayes and McMahon, 2009; Kim *et al*, 2010). More generally, *NRF2* mutations are associated with squamous carcinomas irrespective of the tissue of origin. In one study of 1145 carcinoma samples from 13 tissue types, of which 167 were squamous, 21 of the 22 detected mutations in *NRF2* were in SCC (Kim *et al*, 2010). In a separate integrative genomic and epigenomic analysis of SCC of the lung, somatic alteration of the *CUL3*/*KEAP1*/*NRF2* pathway was detected in 34% of 178 cases (CGARN, 2012). Pertinently, most reports of AKR overexpression in cancer have involved NSCLC. Increased expression of AKR1B and AKR1C isoforms has been demonstrated to occur in both SCC and AC of the lung, but in agreement with our analyses these events are more common in the former subtype (Fukumoto *et al*, 2005; Inamura *et al*, 2005; Woenckhaus *et al*, 2006). Furthermore, our results are consistent with microarray measurements of *AKR1B10* mRNA transcript levels in SCC and AC of the lung (Lee *et al*, 2008; Zhu *et al*, 2010). As AKR1B10 has been shown to be highly upregulated in other types of cancer (Balendiran *et al*, 2009), it would be interesting to determine whether, as with mutation of *NRF2*, increased AKR levels segregate to SCC of tissues other than lung. Future work could also incorporate other AKRs that are known to be regulated by NRF2 but have not been included in this study, such as AKR7A2 (Li *et al*, 2015).

The present work demonstrates that *AKR1B10*, *AKR1C1*, *AKR1C2* and *AKR1C3* are transcriptional targets strongly indicative of NRF2 status in human tumours, and that NRF2 is the upstream effector of AKR overexpression in cancer, particularly in tumours of squamous origin. Although a large number of studies have identified genetic, epigenetic, signalling and other changes that can lead to NRF2 hyperactivation in both AC and SCC of the lung, our data indicate that this is of more widespread downstream consequence in SCC and, moreover, this hyperactivation frequently occurs in the absence of any somatic mutation of the pathway. If, as is widely thought, activation of NRF2 supports tumour growth and proliferation, targeted inhibition of the NRF2 pathway may constitute a potential therapeutic avenue in most cases of this cancer type. In conclusion, therefore, the utilisation of AKRs as biomarkers of NRF2 status will ultimately help determine the significance of this pathway in cancer genesis and progression, and of its potential roles in disease management and the design of therapeutic strategies.

## Figures and Tables

**Table 1:** Immunohistochemical scoring for NSCLC biopsies

**Case**	**AKR1B**	**AKR1C1/2**	**AKR1C3**
AC_1	++	+++	+/−
AC_2	−	−	−
AC_3	−	−	−
AC_4	−	−	−
AC_5	−	−	−
AC_6	−	−	−
AC_7	+/−	+	+
SCC_1	++	+++	+
SCC_2	+	−	++
SCC_3	++	+++	+/−
SCC_4	++	+++	+/−
SCC_5	+	++	−
SCC_6	+/−	++	−
SCC_7	−	+	−
SCC_8	++	+++	−

Abbreviations: AC=adenocarcinoma; SCC=squamous cell carcinoma.

Intensity of IHC staining was scored in seven AC and eight SCC biopsies. −, none detected; +/−, trace; +, weak; ++, moderate, +++, strong.
