# Supplementary material for: Aldo-keto reductases are biomarkers of NRF2 activity and are co-ordinately overexpressed in non-small cell lung cancer
Source: Br J Cancer. 2016 Nov 8;115(12):1530–9. doi: 10.1038/bjc.2016.363 (PMC5155360; doi:10.1038/bjc.2016.363)
Supplement: Supplementary Information [file bjc2016363x1.docx]

**Supplementary data**

Supplementary materials and methods: **Page 2**

Supplementary tables: **Page 5**

Supplementary figures: **Page 10**

*Cell lines*

NCI-H23 and NCI-H522 lines were obtained from the NCI Developmental Therapeutics Program, Division of Cancer Treatment and Diagnosis tumour repository (DTP, Bethesda, MD, USA). NCI-H1395, NCI-H1993, A549, NCI-H460 and NCI-H838 were obtained from the American Type Culture Collection (ATCC, Manassas, VA, USA). EBC-1, HO1-u-1 and LK-2 were obtained from the Japanese Collection of Research Bioresources (JCRB, Osaka, Japan). The above lines were authenticated by the suppliers by STR (DTP and ATCC) or SNP (JCRB) profile and data presented herein are from cells passaged < 10 times in our lab. All other cell lines, including two non-tumour-derived lines for which the *KEAP1* and *NRF2* status are unknown (embryonic kidney AD293 and immortalised keratinocyte HaCaT) were kind gifts from groups within the School of Medicine at the University of Dundee and were authenticated by routine monitoring of morphology and growth rate. All lines were cultured as advised, in either RPMI-1640 (H23, H522, H1395, H1993, H460, H838, LK-2), EMEM (EBC-1), 1:1 DMEM/F-12K (HO1-u-1), F-12K (A549), or DMEM (all other lines), in each case supplemented with 10% foetal bovine serum (media and serum from Life Technologies, Paisley, UK). Cells were grown at 37°C in 5% CO_2_ / 95% air at relative humidity and maintained at <100% confluency with medium replenished every two days and splitting carried out as required (typically twice per week).

*DNA Sequencing*

Cells were trypsinised and genomic DNA purified using the QIAmp DNA Blood Mini kit (Qiagen, Manchester, UK). *KEAP1* coding sequence was amplified by PCR using Q5 high-fidelity DNA polymerase (New England Biolabs, Hitchin, UK), according to manufacturer’s instructions. For NCI-H23, part of exon 2 was amplified using “KEAP1-EX2F2” and “KEAP1-EX2R2” primers, and for NCI-H1395 and NCI-H1993, part of exon 3 was amplified using “KEAP1-EX3F2” and “KEAP1-EX3R2” primers, as per Shibata *et.* *al.* (Shibata *et al*, 2008a). Unused primers and dNTPs were removed from each reaction by Exo-SAP treatment then *KEAP1* sequenced using the PCR primers at the Genetics Core Services Unit, Ninewells Hospital, Dundee.

*Human tissue samples and immunohistochemistry*

Formalin fixed and paraffin embedded (FFPE) samples for immunohistochemistry were selected as a consecutive sample of biopsy-diagnosed non-small cell lung cancer (NSCLC) from clinical samples received at Ninewells Hospital, Dundee, UK. Ethical permission for the use of tissues was approved (Tayside Tissue Bank tissue request number 327) in accordance with the Helsinki Declaration on the use of human tissues for research. Tumour biopsy sections were scored for staining intensity by a qualified pathologist after sample blinding and randomisation.

MCF-7-derived AREc32 cells and A549 cells were fixed in neutral buffered formalin for 24 hours, embedded in 1.5% agarose, then processed into paraffin wax according to standard histological procedures. Sections of these cell pellets were used to determine the optimal immunohistochemical staining method for antigen retrieval and antibody dilution. Following optimization, sections (4 μm) of agarose blocks and patient biopsy samples were stained with rabbit polyclonal anti-AKR1B10, rabbit polyclonal anti-AKR1C1 and mouse monoclonal anti-AKR1C3 using the Avidin-Biotin-Complex peroxidase method (Vector Elite ABC reagents for rabbit or mouse, Vector Laboratories, Peterborough, UK) and avidin/biotin blocking reagents (Vector Laboratories). After de-waxing, endogenous peroxidase activity was blocked by incubation in 0.5% H_2_O_2_ for 35 min at room temperature. Antigen retrieval was performed by boiling for 15 min in 10 mM citrate buffer, pH 6.0, in a microwave oven (for AKR1C1 and AKR1C3; antigen retrieval was omitted for AKR1B10). Sections were incubated in 5% normal goat serum (for rabbit primary antibodies) or 5% normal horse serum (for mouse primary antibody), each containing 5% avidin, for 30 min at room temperature. After washing in PBS, primary antibodies were applied, diluted in 5% normal goat or horse serum containing 5% biotin (AKR1C1: 1/10,000, AKR1C3: 1/2,000, AKR1B10: 1/5,000) at 4°C overnight. Following washes in PBS, biotinylated goat anti-rabbit or horse anti-mouse were applied for 30 min (diluted 1/250) then washed in PBS and incubated with preformed avidin-biotin peroxidase complex. Immunoreactive sites were detected by an intensified DAB reaction, nuclei were counterstained with haematoxylin and sections dehydrated and coverslipped for light microscopy.

| **Cell line** | **Origin** | **Subtype** | **Mutant for** | **Nucleotide change** | **Amino acid change** | **Zygosity** | **Reference** |
| --- | --- | --- | --- | --- | --- | --- | --- |
| EBC-1 | NSCLC | SCC | NRF2 | 230A>T | D77V | heterozygous | Shibata *et al*, 2008b |
| HO-1-u-1 | Oral carcinoma | SCC (oral) | NRF2 | 246A>T | E82D | heterozygous | Shibata *et al*, 2008b |
| H23 | NSCLC | AC | KEAP1 | 579G>C | Q193H | homozygous | Singh *et al*, 2006 |
| A549 | NSCLC | AC | KEAP1 | 997G>T | G333C | homozygous | Singh *et al*, 2006 |
| H460 | NSCLC | large cell carcinoma | KEAP1 | 706G>C | D236H | homozygous | Singh *et al*, 2006 |
| H838 | NSCLC | AC (lymph node metastasis) | KEAP1 | 1330G>T | E444 substitution nonsense | homozygous | Singh *et al*, 2006 |
| LK-2 | NSCLC | SCC | NRF2 | 235G>A | E79K | homozygous | Shibata *et al*, 2008b |

**Supplementary Table 1. *KEAP1* and *NRF2* mutant cell lines used in the study.** For references, see main text.

|  |  |  | ***AKR1B10*** | | ***AKR1C1/2*** | | ***AKR1C3*** | |
| --- | --- | --- | --- | --- | --- | --- | --- | --- |
| **Cell Line** | **Tissue of Origin** | **Genotype (13, 14, 34)** | **SFN** | **TBE-31** | **SFN** | **TBE-31** | **SFN** | **TBE-31** |
| A549 | Lung | *KEAP1* hom | 1.4 ± 0.2 | 1.4 ± 0.5 | 1 ± 0.1 | 0.9 ± 0.1 | 1.1 ± 0.2 | 1 ± 0.1 |
| H460 | Lung | *KEAP1* hom | 1 ± 0 | 1.2 ± 0.5 | 0.6 ± 0.3 | 1 ± 0 | 0.6 ± 0.1 | 0.9 ± 0 |
| H838 | Lung | *KEAP1* hom | 1 ± 0.3 | 1 ± 0.2 | 1.2 ± 0.1 | 0.9 ± 0.3 | 0.7 ± 0.2 | 1.1 ± 0 |
| H23 | Lung | *KEAP1* hom | 2.4 ± 1.4 | 13.9 ± 5.1 | 2.7 ± 0.1 * | 10.4 ± 2.4 * | 0.8 ± 0.1 | 2.1 ± 0.5 |
| LK-2 | Lung | *NRF2* hom | 0.9 ± 0.3 | 1.6 ± 1 | 0.8 ± 0.2 | 1 ± 0.1 | 1.1 ± 0.1 | 1.3 ± 0.1 |
| EBC-1 | Lung | *NRF2* het | 1.7 ± 0.2 | 2.6 ± 0.6 | 3.4 ± 0.6 | 3.4 ± 0.5 * | 1.1 ± 0 | 1.8 ± 0.5 |
| HO1-u-1 | Mouth | *NRF2* het | 1.7 ± 0.3 | 1.6 ± 0.4 | 1.3 ± 0 | 1.3 ± 0.1 | 1.3 ± 0.2 | 1.2 ± 0.1 |
| H522 | Lung | wt | ND | ND | 0.8 ± 0 | 1.3 ± 0.1 | 0.4 ± 0.3 | 0.6 ± 0.5 |
| H1299 | Lung | wt | 9.4 ± 1.2 * | 56.4 ± 13.6 * | 0.8 ± 0 | 2.7 ± 0.4 * | 1.3 ± 0.6 | 14.3 ± 7.9 |
| H1395 | Lung | wt | 7.6 ± 1.2 * | 3.6 ± 0.3 * | 3.4 ± 0.3 * | 1.6 ± 0 * | 1.6 ± 1.2 | 1.1 ± 0 |
| H1993 | Lung | wt | 6 ± 0.9 * | 6.2 ± 1.9 | 4.7 ± 1.1 | 4.5 ± 0.7 * | 3.7 ± 0.6 * | 2.6 ± 0.2 * |
| AD293 | Kidney | unknown | 0.6 ± 0.1 | 5.7 ± 2 | 1.4 ± 0.1 | 2.2 ± 0.7 | 1 ± 0 | 3.1 ± 0.8 |
| A2780 | Ovary | wt | ND | ND | 4.5 ± 1 | 30.1 ± 7.5 * | 1.4 ± 0.2 | 7.6 ± 2.2 |
| OVC433 | Ovary | wt | 3.8 ± 0.2 * | 9.2 ± 1 * | 2.1 ± 0.2 * | 12.1 ± 0 * | 1.8 ± 0.3 | 6.4 ± 0.3 * |
| 5637 | Bladder | wt | 1.3 ± 1.3 | 8.8 ± 3.3 | 1.8 ± 0 * | 6 ± 0.5 * | 1.3 ± 0 | 3.2 ± 0.1 * |
| HeLa | Cervix | wt | 3.4 ± 0.5 * | 6 ± 0.8 * | 16.2 ± 5.6 | 31.6 ± 6 * | 17.4 ± 3.1 * | 35.6 ± 3.6 * |
| A431 | Skin | wt | 4.7 ± 0.3 * | 27.2 ± 2.9 * | 6.3 ± 1 * | 36.9 ± 5.2 * | 5.1 ± 0.8 * | 32.6 ± 4.1 * |
| HaCaT | Skin | unknown | 4.8 ± 0.4 * | 28.9 ± 0.5 * | 12.9 ± 2.6 * | 66 ± 19.1 * | 9.5 ± 0.4 * | 57.8 ± 6.5 * |
| MCF-7 | Breast | wt | 25.9 ± 9.5 | 128.8 ± 26.1 * | 13.4 ± 2.7 * | 41.7 ± 5.5 * | 8.4 ± 2.5 | 27.1 ± 0.9 * |
| MDA-MB-231 | Breast | wt | 6.3 ± 0.3 * | 13.8 ± 2.5 * | 1.5 ± 0.3 | 7.2 ± 0.5 * | 1.8 ± 0.2 | 2.9 ± 0.2 * |
| T47-D | Breast | wt | ND | ND | 3.9 ± 0.6 * | 9.6 ± 0.7 * | 18.1 ± 1.4 * | 56.7 ± 12.4 * |
| HOS | Bone | wt | 4.6 ± 2.1 | 79.4 ± 11.2 * | 0 ± 0 | 0.7 ± 0.2 | 1.9 ± 0.2 | 8.6 ± 2 * |
| U2OS | Bone | wt | 3.4 ± 0.9 | 6.9 ± 0.7 * | 5.5 ± 0.6 * | 30.5 ± 4.6 * | 3.5 ± 0.6 * | 9.6 ± 3.8 |

**Supplementary Table 2. Chemical activators of NRF2 induce AKR mRNA in human cell lines with wild-type *KEAP1* and *NRF2*.** Cells were seeded, incubated under standard conditions for 24 hours, then treated with 5 µmol/L SFN, 0.2 µmol/L TBE-31 or vehicle control. After a further 24 hours, cells were lysed, cDNA synthesised, and RT-PCR carried out for AKR1B10, AKR1C1/2 and AKR1C3. Data are presented as fold change values relative to vehicle control ± SD, and are representative of two separate experiments.

­­

|  |  |  | **AKR1B10** | | **AKR1C1/2** | | **AKR1C3** | | **NQO1** | |
| --- | --- | --- | --- | --- | --- | --- | --- | --- | --- | --- |
| **Cell Line** | **Tissue of Origin** | **Genotype (13, 14, 34)** | **SFN** | **TBE-31** | **SFN** | **TBE-31** | **SFN** | **TBE-31** | **SFN** | **TBE-31** |
| A549 | Lung | *KEAP1* hom | - | - | - | - | - | - | - | - |
| H460 | Lung | *KEAP1* hom | - | - | - | - | - | - | - | - |
| H838 | Lung | *KEAP1* hom | ND | ND | - | - | - | - | - | - |
| H23 | Lung | *KEAP1* hom | ND | ND | ND | ND | ND | ND | - | - |
| LK-2 | Lung | *NRF2* hom | ND | ND | - | - | ND | ND | - | - |
| EBC-1 | Lung | *NRF2* het | ND | ND | ND | ND | ND | ND | - | - |
| HO1-u-1 | Mouth | *NRF2* het | - | - | + | + | - | - | - | - |
| H522 | Lung | wt | ND | ND | ND | ND | ND | ND | ++ | ++ |
| H1299 | Lung | wt | + | + | - | - | ND | ND | ++ | ++ |
| H1395 | Lung | wt | ++ | ++ | + | + | - | - | + | + |
| H1993 | Lung | wt | ND | ND | ND | ND | ND | ND | + | + |
| AD293 | Kidney | unknown | - | - | ND | ND | ND | ND | ND | ND |
| A2780 | Ovary | wt | - | - | - | - | ND | ND | + | + |
| OVC433 | Ovary | wt | + | + | + | ++ | + | ++ | + | + |
| 5637 | Bladder | wt | + | + | ND | ND | - | - | - | - |
| HeLa | Cervix | wt | + | + | ND | ND | ND | ND | - | - |
| A431 | Skin | wt | - | + | ++ | +++ | ++ | +++ | - | + |
| HaCaT | Skin | unknown | ++ | +++ | + | ++ | + | ++ | - | - |
| MCF-7 | Breast | wt | ++ | +++ | + | ++ | + | +++ | + | + |
| MDA-MB-231 | Breast | wt | ND | ND | ND | ND | + | + | ND | ND |
| T47-D | Breast | wt | ND | ND | ND | ND | + | ++ | + | + |
| HOS | Bone | wt | - | + | ND | ND | ND | ND | - | - |
| U2OS | Bone | wt | - | - | + | + | + | +++ | - | - |

**Supplementary Table 3. Chemical activators of NRF2 induce AKR protein in human cells with wild-type *KEAP1* and *NRF2*.** Cells were seeded, incubated under standard conditions for 24 hours, then treated with 5 µmol/L SFN, 0.2 µmol/L TBE-31 or vehicle control. After a further 24 hours, cells were lysed and protein samples immunoblotted for AKR1B10, AKR1C1/2, AKR1C3 and NQO1. ND: not detectable, “-“: no change, “+”: weak induction, “++”: moderate induction, “+++”: strong induction.

| ***AKR1B10*** | ***AKR1C1*** | ***AKR1C2*** | ***AKR1C3*** |
| --- | --- | --- | --- |
| **TCGA-05-4382**  *KEAP1*(HOMDEL) | **TCGA-05-4382**  *KEAP1*(HOMDEL) | **TCGA-05-4382**  *KEAP1*(HOMDEL) | **TCGA-05-4382** *KEAP1*(HOMDEL) |
| **TCGA-05-4415**  *KEAP1*(HOMDEL,DOWN) | **TCGA-05-4415**  *KEAP1*(HOMDEL,DOWN) | **TCGA-05-4415**  *KEAP1*(HOMDEL,DOWN) | **TCGA-05-4415**  *KEAP1*(HOMDEL,DOWN) |
| **TCGA-55-6982**  *NRF2*(MUT) | **TCGA-50-5936**  *KEAP1*(MUT) | **TCGA-50-6673**  *NRF2*(AMP,UP) | **TCGA-50-5936**  *KEAP1*(MUT) |
| **TCGA-55-7815**  *KEAP1*(MUT,DOWN) | **TCGA-50-6673**  *NRF2*(AMP,UP) | **TCGA-55-6982**  *NRF2*(MUT) | **TCGA-50-6673**  *NRF2*(AMP,UP) |
| **TCGA-55-7907**  *KEAP1*(HOMDEL) | **TCGA-55-6982**  *NRF2*(MUT) | **TCGA-55-7907**  *KEAP1*(HOMDEL) | **TCGA-55-6982**  *NRF2*(MUT) |
| **TCGA-73-4658**  *NRF2*(MUT) | **TCGA-55-7907**  *KEAP1*(HOMDEL) | **TCGA-73-4658**  *NRF2*(MUT) | **TCGA-55-7907**  *KEAP1*(HOMDEL) |
| **TCGA-73-7498**  *KEAP1*(MUT) | **TCGA-73-4658**  *NRF2*(MUT) | **TCGA-78-7158**  *KEAP1*(MUT) | **TCGA-73-4658**  *NRF2*(MUT) |
| **TCGA-78-7158**  *KEAP1*(MUT) | **TCGA-78-7158**  *KEAP1*(MUT) | **TCGA-95-7039**  *NRF2*(AMP,UP) | **TCGA-78-7158**  *KEAP1*(MUT) |
| **TCGA-78-7633**  *KEAP1*(MUT) | **TCGA-95-7039**  *NRF2*(AMP,UP) | **TCGA-95-7947**  *KEAP1*(MUT) | **TCGA-95-7039**  *NRF2*(AMP,UP) |
| **TCGA-95-7039**  *NRF2*(AMP,UP) | **TCGA-95-7947**  *KEAP1*(MUT) | **TCGA-97-7941**  *NRF2*(MUT) | **TCGA-95-7947**  *KEAP1*(MUT) |

**Supplementary Table 4. A shared signature of low *AKR* expression between TCGA cases of *KEAP1*/*NRF2* mutant AC.** TCGA case ID numbers for the ten samples in which expression of each AKR is lowest are shown in bold. Those cases which are present in two or more columns are coded with the same colour. HOMDEL: homozygous deletion, MUT: mutation, AMP: amplification, UP: upregulation, DOWN: downregulation.

| *AKR1B10* | | *AKR1C1* | | *AKR1C2* | | *AKR1C3* | |
| --- | --- | --- | --- | --- | --- | --- | --- |
| Gene symbol | Spearman score | Gene symbol | Spearman score | Gene symbol | Spearman score | Gene symbol | Spearman score |
| *AKR1C3* | 0.84 | *AKR1C3* | 0.95 | *AKR1C4* | 0.96 | *AKR1C4* | 0.97 |
| *SRXN1* | 0.83 | *AKR1C4* | 0.94 | *AKR1C3* | 0.95 | *AKR1C2* | 0.95 |
| *AKR1C1* | 0.83 | *AKR1C2* | 0.94 | *AKR1C1* | 0.94 | *AKR1C1* | 0.95 |
| *CYP4F3* | 0.81 | *CYP4F3* | 0.84 | *TALDO1* | 0.81 | *SRXN1* | 0.84 |
| *AKR1C4* | 0.8 | *OSGIN1* | 0.84 | *GPX2* | 0.8 | *ME1* | 0.84 |
| *AKR1C2* | 0.8 | *PGD* | 0.83 | *CYP4F3* | 0.8 | *AKR1B10* | 0.84 |
| *PGD* | 0.8 | *TALDO1* | 0.83 | *AKR1B10* | 0.8 | *TRIM16L* | 0.83 |
| *CYP4F11* | 0.8 | *AKR1B10* | 0.83 | *SRXN1* | 0.79 | *OSGIN1* | 0.83 |
| *OSGIN1* | 0.8 | *CYP4F11* | 0.83 | *PGD* | 0.79 | *TALDO1* | 0.82 |
| *GCLM* | 0.78 | *SRXN1* | 0.82 | *CYP4F11* | 0.79 | *NQO1* | 0.81 |
| *ME1* | 0.78 | *TRIM16L* | 0.82 | *OSGIN1* | 0.79 | *GPX2* | 0.81 |
| *TALDO1* | 0.78 | *GPX2* | 0.81 | *ME1* | 0.78 | *CYP4F3* | 0.81 |
| *TXN* | 0.78 | *ME1* | 0.81 | *TRIM16L* | 0.78 | *PGD* | 0.81 |
| *CBR3* | 0.77 | *GCLC* | 0.8 | *TXN* | 0.78 | *TXN* | 0.81 |
| *PTGR1* | 0.77 | *RIT1* | 0.8 | *NQO1* | 0.77 | *CYP4F11* | 0.81 |
| *ADH7* | 0.76 | *ADH7* | 0.79 | *RIT1* | 0.77 | *GCLC* | 0.78 |
| *G6PD* | 0.76 | *TXN* | 0.79 | *GCLC* | 0.76 | *RIT1* | 0.78 |
| *TRIM16L* | 0.76 | *CYP4F2* | 0.79 | *UGT1A1* | 0.76 | *ADH7* | 0.77 |
| *ALDH3A1* | 0.75 | *TMEM116* | 0.79 | *UGT1A3* | 0.76 | *ALDH3A1* | 0.77 |
| *GPX2* | 0.75 | *SLC7A11* | 0.79 | *UGT1A4* | 0.76 | *G6PD* | 0.77 |
| *RIT1* | 0.75 | *UGT1A1* | 0.79 | *UGT1A9* | 0.76 | *SLC7A11* | 0.77 |
| *CYP4F2* | 0.75 | *UGT1A3* | 0.79 | *UGT1A8* | 0.76 | *UGT1A1* | 0.77 |
| *CES1* | 0.74 | *UGT1A4* | 0.79 | *UGT1A7* | 0.76 | *UGT1A3* | 0.77 |
| *NQO1* | 0.74 | *UGT1A9* | 0.79 | *UGT1A6* | 0.76 | *UGT1A4* | 0.77 |
| *SLC7A11* | 0.74 | *UGT1A8* | 0.79 | *UGT1A5* | 0.76 | *UGT1A9* | 0.77 |
| *EPHX1* | 0.72 | *UGT1A7* | 0.79 | *ADH7* | 0.75 | *UGT1A8* | 0.77 |
| *ANXA10* | 0.72 | *UGT1A6* | 0.79 | *ALDH3A1* | 0.75 | *UGT1A7* | 0.77 |
| *TRIM16* | 0.72 | *UGT1A5* | 0.79 | *G6PD* | 0.75 | *UGT1A6* | 0.77 |
| *GSR* | 0.71 | *NQO1* | 0.78 | *UGT1A10* | 0.75 | *UGT1A5* | 0.77 |
| *PRDX1* | 0.71 | *G6PD* | 0.78 | *CYP4F2* | 0.74 | *GCLM* | 0.76 |

**Supplementary Table 5. Co-expression analysis of mRNA in SCC.** Cases of SCC (n=178) were assessed for co-expressed genes using cBioPortal. Spearman’s rank correlation coefficients are shown for the 30 most highly positively correlated transcripts with each of *AKR1B10*, *AKR1C1*, *AKR1C2* and AKR1C3.

**
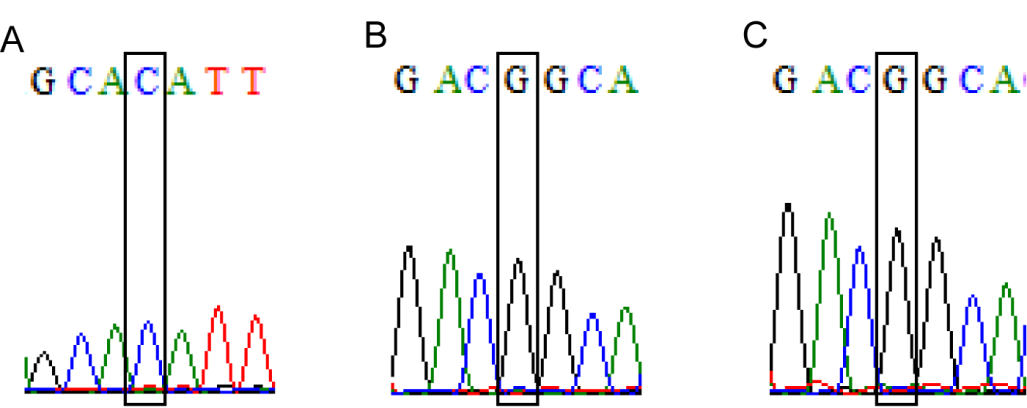
**

**Supplementary Figure 1. Sequencing of *KEAP1* in cultured cell lines.** (A) The H23 cell line contains a 579G>C point mutation, encoding a Q193H codon change. (B) The H1395 cell line does not contain a 1048G>A point mutation. (C) The H1993 cell line does not contain a 1048G>A point mutation.

**
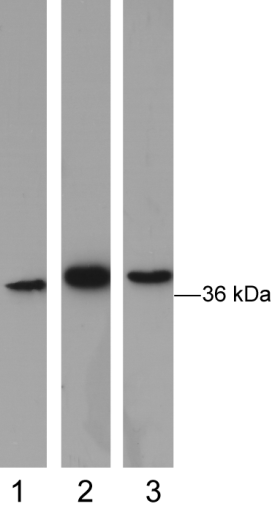
**

**Supplementary Figure 2. IHC method development: antibody specificity.** Western blotting using AKR antibodies gives a single band in samples from A549 cells at the predicted molecular weight. 1: anti-AKR1B, 2: anti-AKR1C1/2, 3: anti-AKR1C3.

**
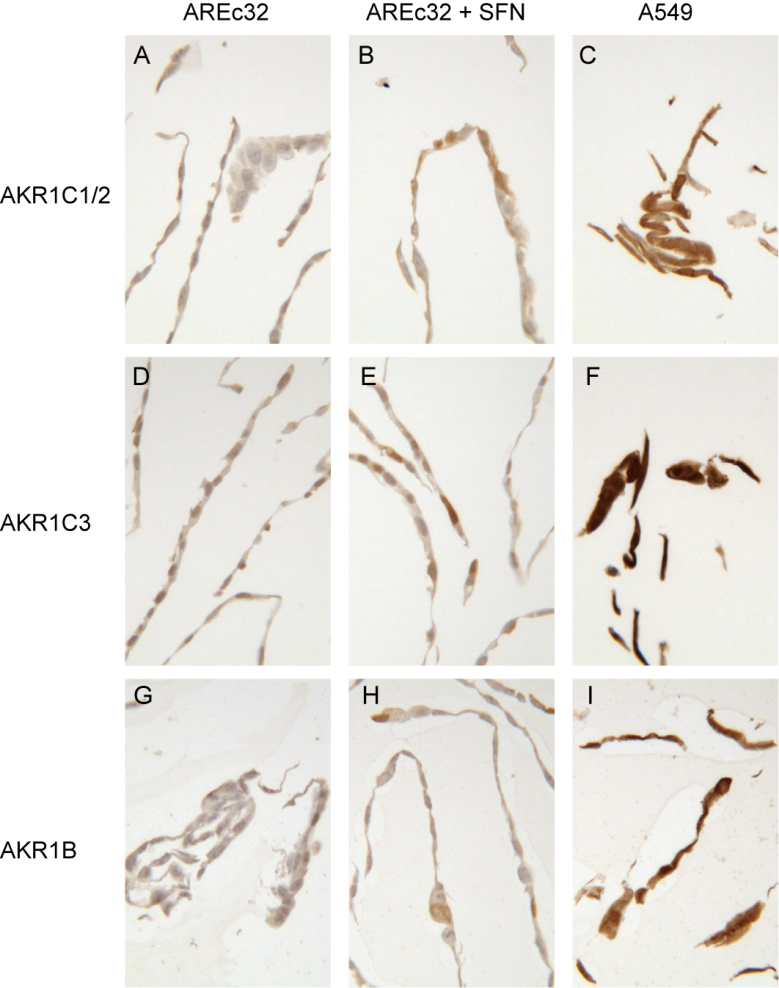
**

**Supplementary Figure 3. IHC method development: antibody optimisation in cell lines.** Immunohistochemistry with anti-AKR1C1/2 in (A) AREc32, (B) AREc32 + SFN and (C) A549 cells, anti-AKR1C3 in (D) AREc32, (E) AREc32 + SFN and (F) A549 cells, and anti-AKR1B in (G) AREc32, (H) AREc32 + SFN and (I) A549 cells.

**
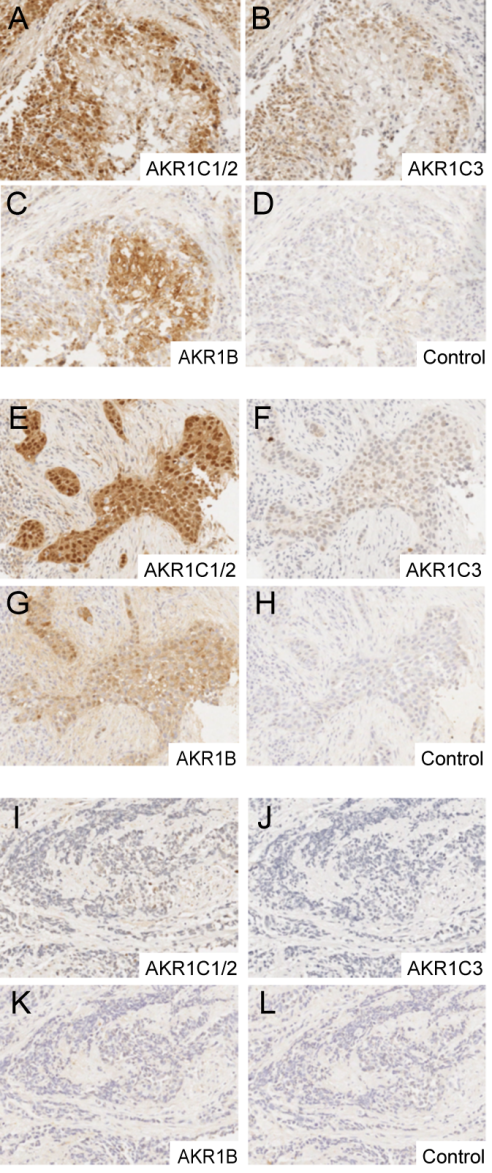
**

**Supplementary Figure 4. Representative images of AKR expression in SCC and AC.**

Representative immunohistochemical images of two separate SCC (A-D and E-H) and one AC (I-L) are shown, stained for AKR1C1/2 (A, E, I), AKR1C3 (B, F, J), AKR1B (C, G, K), and without primary antibody (negative control; D, H, L).


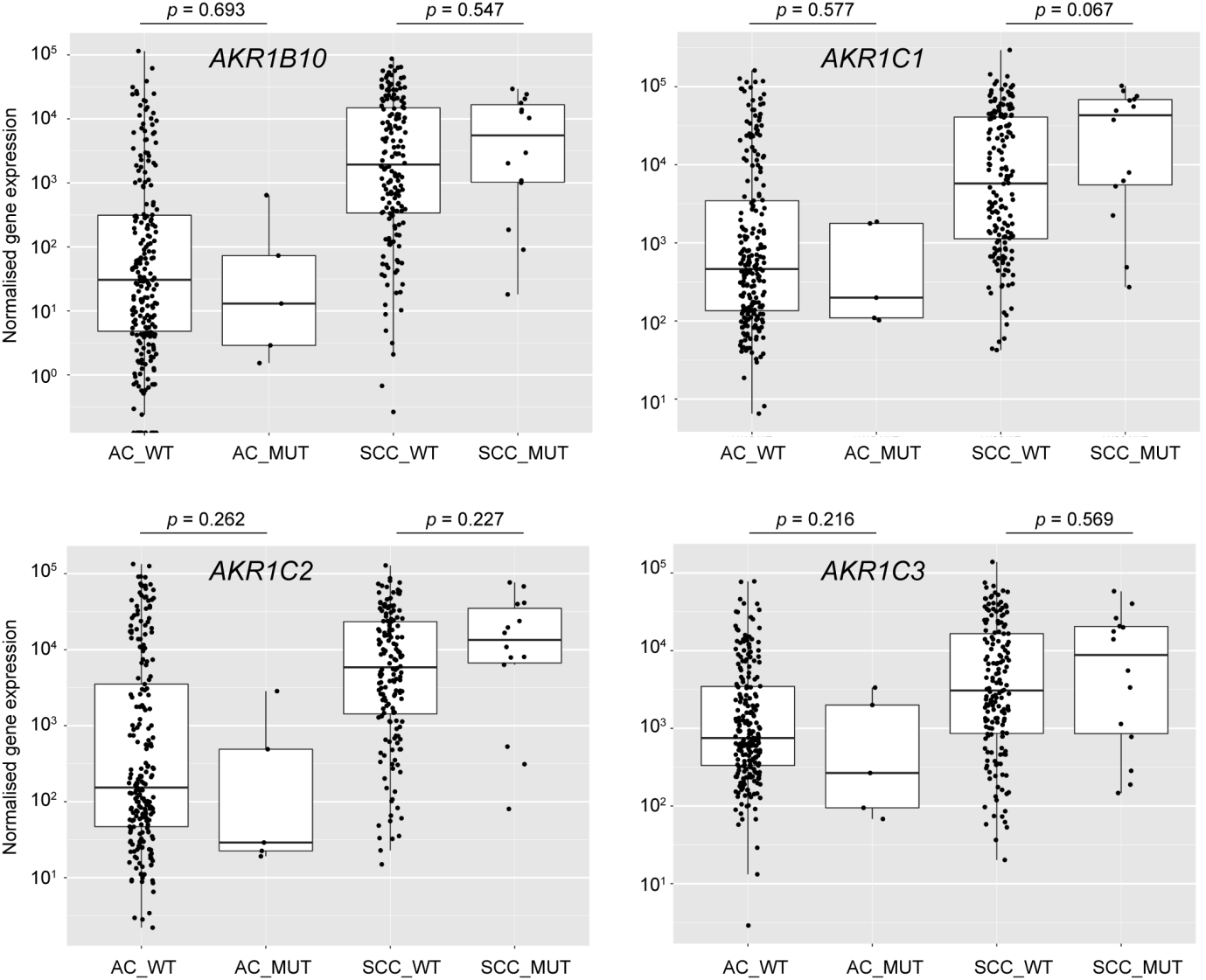


**Supplementary Figure 5. *AKR* mRNA levels in *CUL3* mutated AC and SCC.** Both AC and SCC cases were defined as mutant (MUT) if they possessed somatic mutation and/or loss of heterozygosity of *CUL3*. Association was evaluated by unpaired Wilcoxon ranked test.


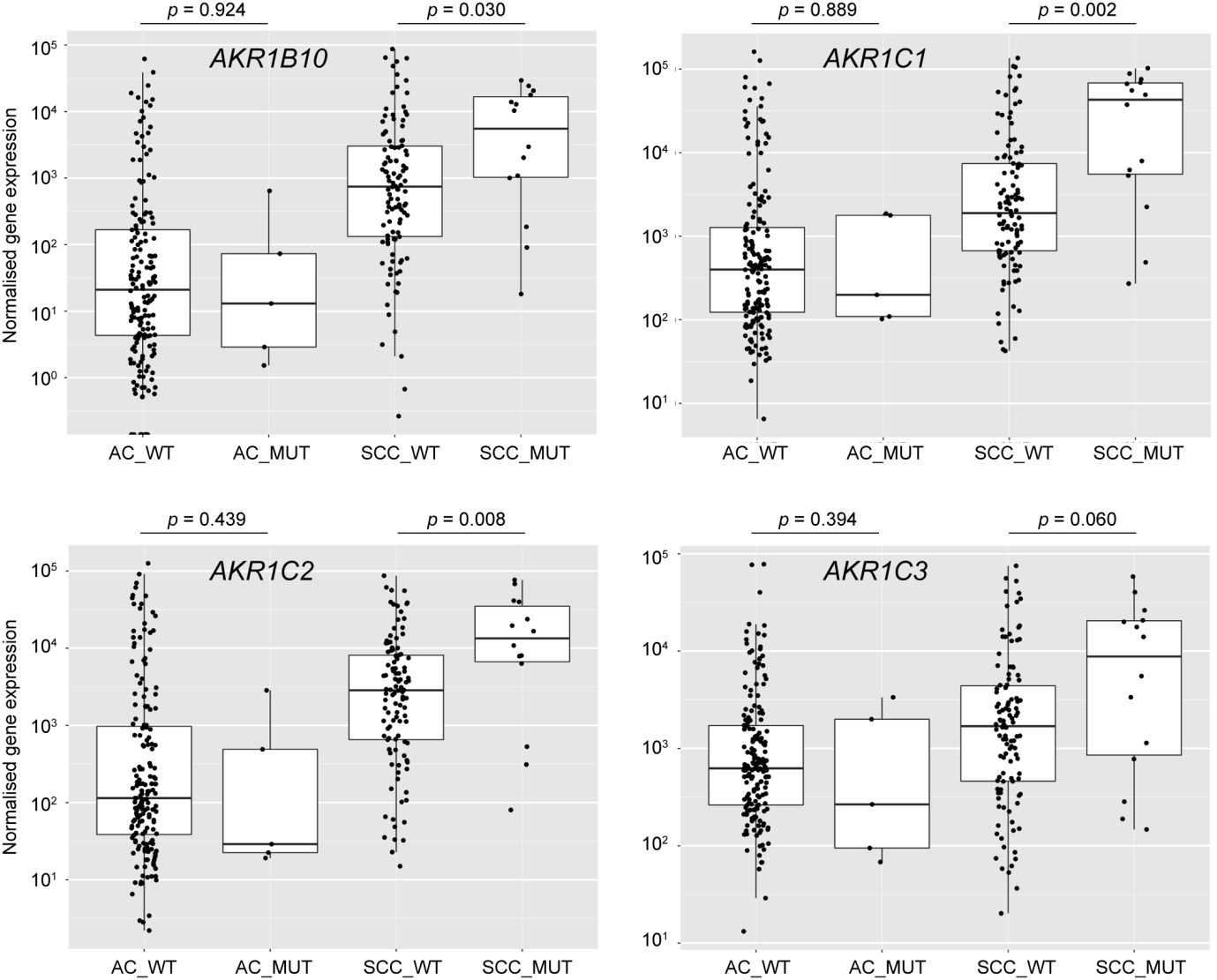


**Supplementary Figure 6. *AKR* mRNA levels in *CUL3* mutated AC and SCC after removal of *NRF2*/*KEAP1* mutant cases.** Both AC and SCC cases were defined as mutant (MUT) if they possessed somatic mutation and/or loss of heterozygosity of *CUL3*. Cases which possessed one or more of the following were removed from the WT groups: somatic mutation of *KEAP1*, loss of heterozygosity of *KEAP1*, somatic mutation of *NRF2*, gene amplification of *NRF2.* Association was evaluated by unpaired Wilcoxon ranked test.


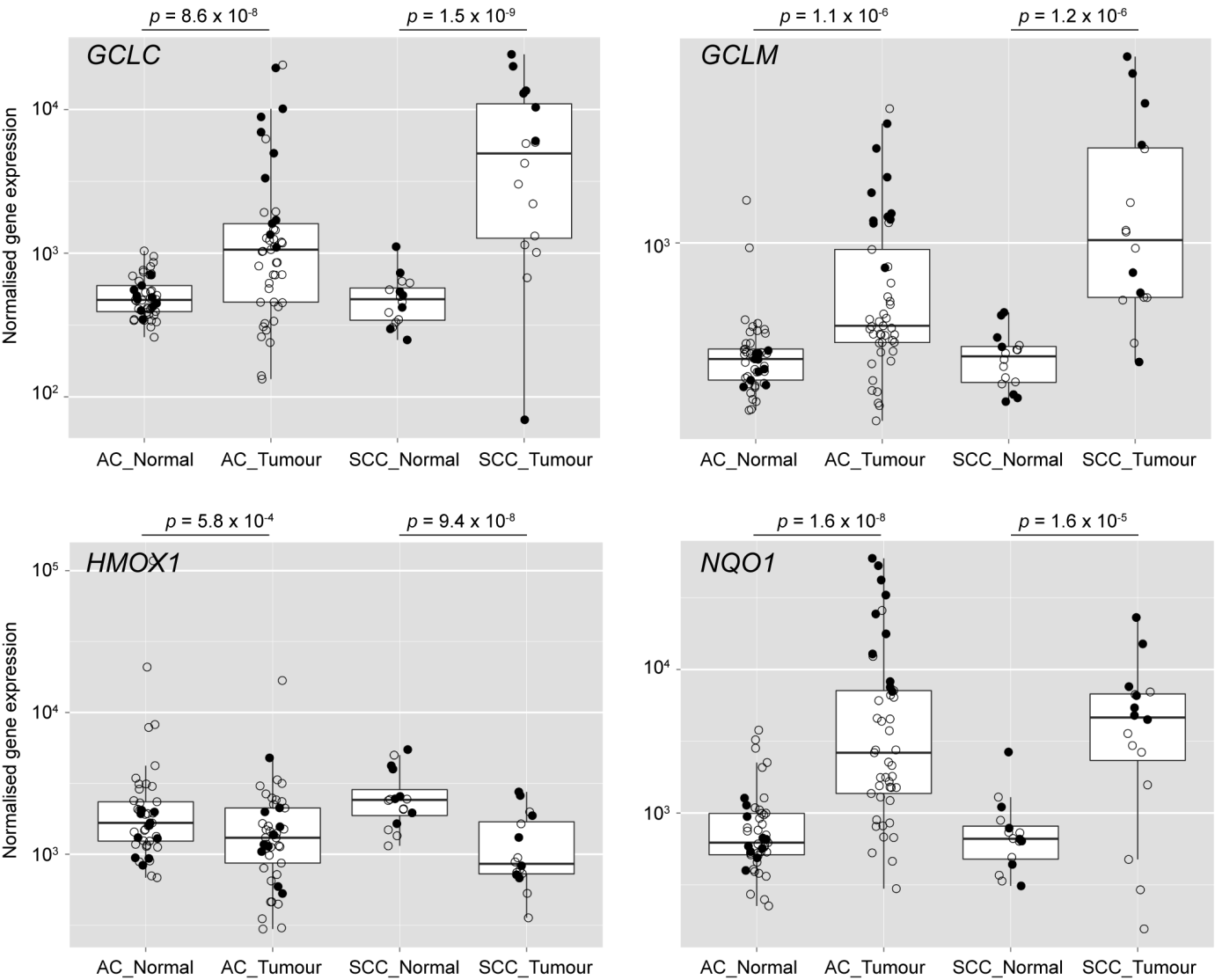


**Supplementary Figure 7. Induction of mRNA for other mRNA target genes in AC and SCC.** Paired normal/tumour sample data from TCGA were processed and analysed as described in Materials and Methods. Cases in which the tumour was either wild-type (open circles) or mutant (closed circles) in respect to *KEAP1*/*NRF2* mutation status are shown. Statistical significance of *AKR* enrichment relative to normal tissue is calculated as a combined score for both wild-type and mutant cases. Association was evaluated by paired Wilcoxon ranked test.


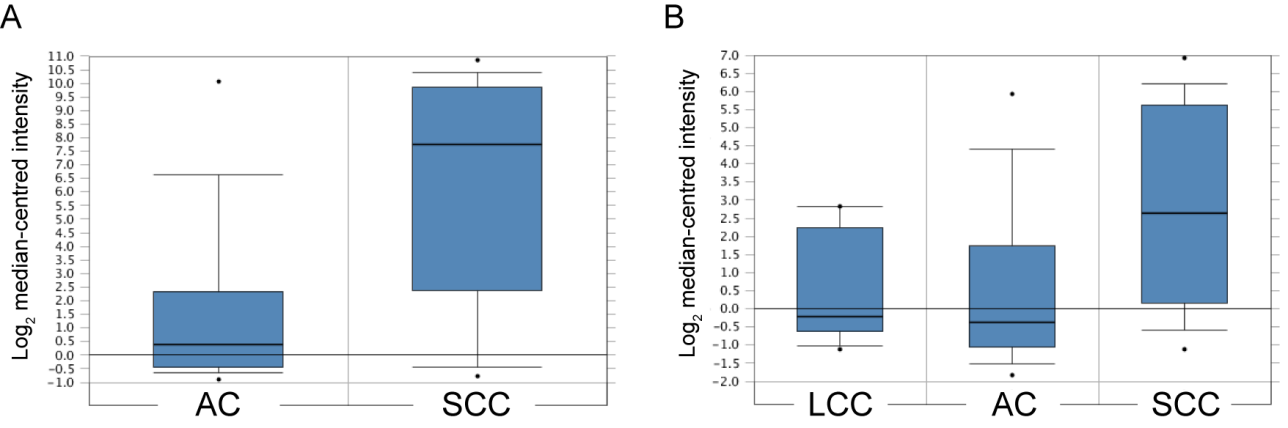


**Supplementary Figure 8. AKR1B10 mRNA expression in lung cancer**. Date were obtained using the Oncomine transcriptome analysis platform. Values shown are log_2_ median-centred intensity of array-normalised data. (A) 1: AC (n=63), 2: SCC (n=75, *p*=1.29x10^-12^) (23). (B) 1: Large cell carcinoma (LCC, n=10), 2: AC (n=28), 3: SCC (n=52, *p*=2.63x10^-6^) (24).


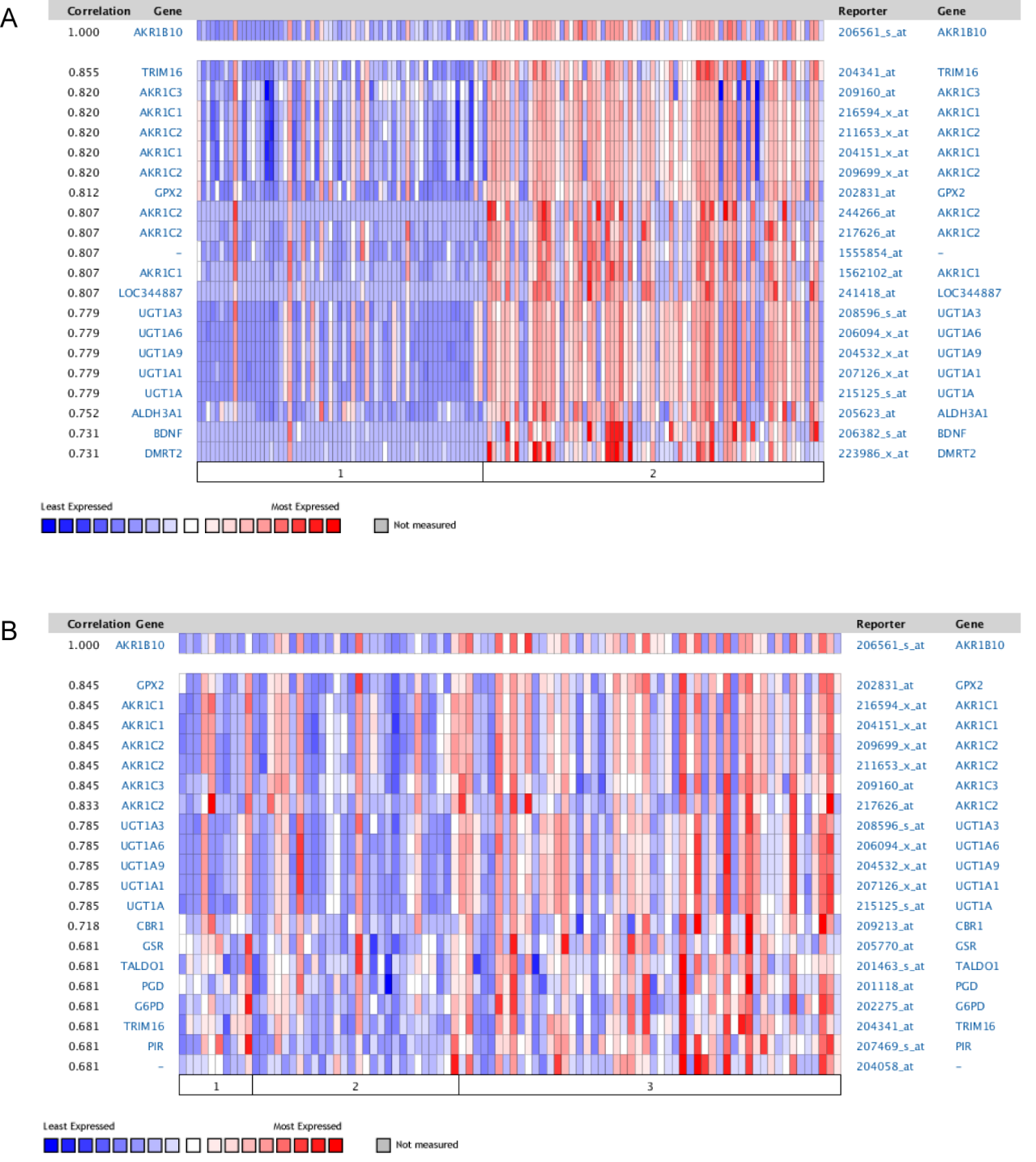


**Supplementary Figure 9. Co-expression analysis with AKR1B10 mRNA in lung cancer**. Data were obtained using the Oncomine transcriptome analysis platform. Correlation values, relative to AKR1B10, are derived from average linkage hierarchical clustering, across all samples, as described at www.oncomine.org. The most strongly correlating targets are shown, with a higher value reflecting a stronger correlation. Blue boxes denote a low level of expression and red boxes denote a high level of expression (normalised within rows). (A) 1: Non-small cell lung carcinoma (n=63), 2: SCC (n=75) (23) (B) 1: Large cell carcinoma (n=10), 2: AC (n=28), 3: SCC (n=52) (24).
